# The genetic architecture of socially-affected traits: a GWAS for direct and indirect genetic effects on survival time in laying hens showing cannibalism

**DOI:** 10.1186/s12711-018-0409-7

**Published:** 2018-07-23

**Authors:** Tessa Brinker, Piter Bijma, Addie Vereijken, Esther D. Ellen

**Affiliations:** 1 Animal Breeding and Genomics, Wageningen University and Research, P.O. Box 338, 6700 AH Wageningen, The Netherlands; 2 Research and Technology Centre, Hendrix Genetics, P.O. Box 114, 5830 AC Boxmeer, The Netherlands

## Abstract

**Background:**

Cannibalism is an important welfare problem in the layer industry. Cannibalism is a social behavior where individual survival is affected by direct genetic effects (DGE) and indirect genetic effects (IGE). Previous studies analysed repeated binomial survival, instead of survival time, which improved accuracies of breeding value predictions. Our study aimed at identifying SNPs associated with DGE and IGE for survival time, and comparing results from models that analyse survival time and repeated binomial survival.

**Methods:**

Survival data of three layer crosses (W1 * WA, W1 * WB, and W1 * WC) were used. Each individual had one survival time record and 13 monthly survival (0/1) records. Approximately 30,000 single nucleotide polymorphisms (SNPs) were included in the genome-wide association study (GWAS), using a linear mixed model for survival time, a linear mixed model and a generalized linear mixed model for repeated binomial survival (0/1). Backwards elimination was used to determine phenotypic and genetic variance explained by SNPs.

**Results:**

The same quantitative trait loci were identified with all models. A SNP associated with DGE was found in cross W1 * WA, with an allele substitution effect of 22 days. This SNP explained 3% of the phenotypic variance, and 36% of the total genetic variance. Four SNPs associated with DGE were found in cross W1 * WB, with effects ranging from 16 to 35 days. These SNPs explained 1 to 6% of the phenotypic variance and 9 to 44% of the total genetic variance. Our results suggest a link of DGE and IGE for survival time in layers with the gamma-aminobutyric acid (GABA) system, since a SNP located near a gene for a GABA receptor was associated with DGE and with IGE (not significant).

**Conclusions:**

This is one of the first large studies investigating the genetic architecture of a socially-affected trait. The power to detect SNP associations was relatively low and thus we expect that many effects on DGE and IGE remained undetected. Yet, GWAS results revealed SNPs with large DGE and a link of DGE and IGE for survival time in layers with the GABAergic system, which supports existing evidence for the involvement of GABA in the development of abnormal behaviors.

**Electronic supplementary material:**

The online version of this article (10.1186/s12711-018-0409-7) contains supplementary material, which is available to authorized users.

## Background

Mortality due to cannibalism has important welfare and economic implications in the commercial laying hen industry. Brinker et al. [1] reported mortality to be between 22 and 37% in crossbred chickens with intact beaks, while beak-trimmed hens of the same crosses showed a mortality of 2 to 3% at the end of the laying period (personal communication J. Visscher), which indicates that a substantial part of the mortality is due to cannibalism. Previous research revealed that survival time is affected both by an individual’s own genes (direct genetic effects; DGE) and by genes of its group mates (indirect genetic effects; IGE). It was found that IGE contribute 33 to 76% of the heritable variation in survival time in purebred and crossbred laying hens with intact beaks [2, 3]. However, the genetic architecture of survival time in laying hens that show cannibalism remains largely unknown.

The availability of genomic information has increased our understanding of complex traits, but studies have mainly focussed on DGE. Results from genome-wide association studies (GWAS) on DGE show that most quantitative traits in livestock are highly polygenic and that variants tend to be associated with more than one trait [4]. However, the genetic architecture of IGE may differ from the genetic architecture of DGE. For example, IGE are less exposed to natural selection compared to DGE [5], and therefore we expect that some loci may have large effects for IGE. A few studies have investigated the genetic architecture of IGE. Biscarini et al. [6] conducted an association study using 1022 single nucleotide polymorphisms (SNPs) and identified 81 SNPs that were associated with IGE for plumage condition in laying hens. However, the number of observations used was limited; 662 laying hens originating from nine lines were used for analyses. Mutic and Wolf [7] identified 13 quantitative trait loci (QTL) for IGE associated with size, development, and fitness related traits in *Arabidopsis*. To increase the power of IGE detection, they did not consider loci that did not have DGE in their IGE analyses.

From a statistical point of view, survival time is a difficult trait because many laying hens are still alive at the end of the recording period. For these hens, true survival time cannot be observed but is known to exceed the length of the recording period (censored). Several statistical techniques have been proposed to deal with survival data and IGE, including survival time analysis [8, 9] and the use of repeated binomial survival records (0/1) [10]. Ellen et al. [9] showed that survival time analysis did not improve breeding value predictions compared to analysing survival time with an ordinary mixed linear model when censoring occurs at the same moment in time. Compared to a linear mixed model analysis of survival time, the use of repeated binomial survival records (0/1) by Brinker et al. [10] improved accuracies of breeding value predictions up to 21%. Hence, the use of repeated binomial survival records (0/1) may also be beneficial for the identification of direct and indirect SNP associations in GWAS.

This study had two aims: (1) to identify SNPs associated with direct and indirect effects for survival time in laying hens that show cannibalism; and (2) to compare GWAS results from analysis of survival time versus repeated binomial survival (0/1).

## Methods

### Genetic stock and pedigree

Data were collected under the control of Hendrix Genetics. Hendrix Genetics complies with the Dutch law on animal welfare. Hendrix Genetics provided data on three crossbred White Leghorn layer lines. Crossbreds descended from one sire line (W1) and three dam lines (WA, WB, and WC), and were coded W1 * WA, W1 * WB, and W1 * WC. In contrast to Brinker et al. [1], data from cross W1 * WD were not used in this study because the quality of the genomic data was insufficient.

A total of 159 sires and 3218 dams were used, with 48 to 57 sires per cross. For each cross, matings between sires and dams were randomly assigned, which resulted in approximately two female offspring per dam. The sire pedigree was recorded for all offspring. The dam’s pedigree was initially unknown but a reconstructed pedigree, based on genomic information, was provided by Hendrix Genetics.

### Housing conditions

Chickens of the three crosses hatched simultaneously in the Netherlands. One-day old chickens were vaccinated, wing-banded, and transported to Canada. Chickens had intact beaks. Chickens were transported to a laying house at approximately 17 weeks of age. The laying house consisted of two wings. In each wing, rows were grouped into six double rows that each contained two levels, with a corridor between each double row to allow access to cages. Each cage contained five individuals of the same sire. Thus, cage mates were either paternal half-sibs or full-sibs. A standard commercial layer diet and water were provided ad libitum. A feeding trough was in front of the cages and each cage had its own drinking nipples. The light intensity was stronger at the top level compared to the bottom level, due to closer proximity to light sources. Further details are in Brinker et al. [1].

### Data

Dead hens were removed daily but the cause of death was not determined. The wing band number and cage number were recorded after death. The study was terminated when hens were approximately 75 weeks old. Survival at the end of the study was 78, 75, and 63%, for crosses W1 * WA, W1 * WB, and W1 * WC, respectively.

Cages with initially less than five hens and cages with mistakes in their composition (*e.g.* with hens descending from multiple sires) were removed from the dataset. Cages with hens descending from multiple sires were identified based on genomic information.

Survival time was defined as the number of days from the start of the laying period until either death or the end of the study, with a maximum of 402 days. Hens that were alive at the end of the laying period were given a survival time of 402 days. In total, records on 1920, 1875, and 1620 laying hens were used for the statistical analyses for crosses W1 * WA, W1 * WB, and W1 * WC, respectively (Table 1).

**Table 1:** Data description of three crossbred layer lines, number of crossbreds phenotyped and genotyped, and number of SNPs after quality control

Sire line	Dam line	Mean survival days ± SD	Number of phenotypes		Number of genotyped crossbreds	Number of SNPs
			Survival time	Survival (0/1)		
W1	WA	364.6 ± 87.9	1920	24,960	1889	27,204
W1	WB	349.8 ± 107.0	1875	24,375	1816	32,473
W1	WC	323.9 ± 123.2	1620	21,060	1580	38,588

To generate repeated binomial survival records (0/1), the laying period was divided into 13 months. For each month, survival was coded 1 if the laying hen was alive at the end of that month and as 0 if not. Thus, a survival record (0/1) was available for each month. In total, 24,960, 24,375, and 21,060 monthly records were available for crosses W1 * WA, W1 * WB, and W1 * WC respectively (Table 1).

### Genotyping and SNP quality

Birds were genotyped based on DNA extracted from blood, using a custom made Illumina 60 K chicken SNP BeadChip, which included 52,232 SNPs across chromosomes 1 through 28, Z, W, and two unmapped linkage groups, along with some unassigned SNPs. PLINK [11, 12] was used for the quality control of genotypes. SNPs with a missing rate higher than 0.30 and a MAF lower than 0.005 were removed. Individuals with a missing rate higher than 0.10 were also removed. SNPs that deviated from Hardy–Weinberg Equilibrium (*p* < 10^−5^) in the parental population were removed from the full dataset. SNPs contributing more than one Mendelian error and individuals contributing more than five Mendelian errors were also removed from the dataset. Remaining Mendelian errors were set to missing.

The number of genotyped crossbreds and the number of SNPs available after quality control for each cross are in Table 1. Some individuals were not genotyped (max. 4%) due to death before blood sampling (~ 1 month after the start of the laying period) or due to poor DNA quality. To allow cages that contained individuals without genotype information to be included in the statistical analyses, missing genotypes were replaced by the “parental mean”, which was the average of the allele count of the sire and the average allele count of its mates (~ 20 dams). In case parental genotypes were missing, the line average was used. The same procedure was applied for missing genotype information, *i.e.* for genotypes associated with Mendelian errors.

### Statistical analysis

Data were analyzed separately for each cross. Three statistical models were compared: a linear mixed model for survival time (STM), a linear mixed model for repeated binomial survival (0/1; RMM.t), and a generalized linear mixed model for repeated binomial survival (0/1; GLMM). All models were implemented using ASReml [13].

First, genetic parameters were estimated without SNP effects in the model (see models below). Five generations of (reconstructed) pedigree information on sires and dams were included in all genetic analyses. In our data, cages consisted of paternal half-sibs, with an occasional full-sib. Therefore, direct and indirect polygenic effects were strongly confounded [1, 14]. With cages composed of families, an animal model with DGE only will also pick up IGE and consequently yields genetic parameter estimates that refer to the total breeding value [14]. Thus, an animal model with DGE only was used to account for population stratification (*i.e.* family structure in the population). This animal model incorporated the genetic covariance structure across individuals, to avoid spurious SNP associations due to relatedness.

Second, SNP effects were estimated one by one, including both the direct SNP effect of the individual and the summed indirect SNP effects of its cage mates in the model. For all models, variance components were fixed to the estimated values from the corresponding model without the SNP effect. Direct and indirect SNP effects were fitted simultaneously, because a GWAS with direct SNP effects only would also capture part of the indirect effect of the SNP, as the related group mates have an above-average probability to carry the same alleles.

#### Survival time model—STM

Survival time records were analysed using the following linear mixed model:

1 $$\begin{aligned} y_{{ik}} & = fixed + \beta _{D} \cdot SNP_{i} + \beta _{I} \cdot \sum\limits_{{j \ne i}}^{{n - 1}} S NP_{{j\left( k \right)}} \\ & \quad + \;a_{i} + cage_{k} + e_{{ijk}} , \\ \end{aligned}$$

where $y_{ik}$ is the observed survival time (days) for individual $i$ in cage $k$, with cage mates $j$, $n$ is the number of cage members ($n$= 5), and *fixed* represents the fixed effect of the combination of wing-row-level; $\beta_{D}$ and $\beta_{I}$ are regression coefficients of $y_{ik}$ on SNP genotypes fitted as fixed effects, where $\beta_{D}$ is the fixed direct effect of the SNP of individual $i$, and $\beta_{I}$ is the fixed indirect effect of the same SNP in cage mates $j$; $SNP_{i}$ is the allele count (0, 1, 2) for the individual, $\mathop \sum \nolimits_{j \ne i}^{n - 1} SNP_{j\left( k \right)}$ is the summed SNP allele counts (0–8) of cage mates $j$, $a_{i}$ is the random polygenic effect of individual $i$, $cage_{k}$ is the random cage effect, and $e_{ijk}$ is the residual. All random effects were assumed to be normally distributed. The covariance structures for the model terms were: $var\left( a \right) = {\mathbf{A}}\sigma_{a}^{2}$, $var\left( {cage} \right) = {\mathbf{I}}\sigma_{cage}^{2}$, and $var\left( e \right) = {\mathbf{I}}\sigma_{e}^{2}$, where ${\mathbf{A}}$ is a pedigree relationship matrix, $\sigma_{a}^{2}$ is the additive genetic variance, ${\mathbf{I}}$ is an identity matrix, $\sigma_{cage}^{2}$ is the cage variance, and $\sigma_{e}^{2}$ is the residual variance.

#### Repeated measures model RMM.t

Repeated binomial records on survival (0/1) were analysed using a repeated measures model that included random regressions on time (hence RMM.t). Following [10], the model was:

2 $$\begin{aligned} y_{ikm} = & fixed + \beta_{D} \cdot {\text{t}}_{m} \cdot SNP_{i} + \beta_{I} \cdot {\text{t}}_{m} \cdot \mathop \sum \limits_{j \ne i}^{n - 1} SNP_{j\left( k \right)} \\ \quad +\, a_{i} \cdot {\text{t}}_{m} + cage_{km} + cage_{k} \cdot t_{m} + PE_{i} \cdot t_{m} + e_{ijkm} , \\ \end{aligned}$$

where $y_{ikm}$ is the observed survival (0/1) for individual $i$ in cage $k$ and month $m$, with cage mates $j$, at time $t_{m}$ measured in months since the start of the experiment; *fixed* represents the interaction effect of wing-row-level with time, which was a sixth-order polynomial of time, fitted as a fixed effect, which was used to model the survival curve across time and to allow this curve to depend on location (i.e., on the wing-row-level combination); $cage_{km}$ is the random effect of cage $k$ at time $m$, which accounts for covariances between cage members at specific time points; $cage_{k}$ is the random permanent effect of cage $k$, with $cage_{k} \cdot t_{m}$ accounting for covariances between records on the same cage at different time points, and for increasing variance over time. Together, the $cage_{km}$ and $cage_{k}$ effects account for similarity of cage mates due to shared environment, which is essential to avoid inflation of genetic estimates in the analysis of socially-affected traits [15]. Finally, $PE_{i}$ is the random permanent environmental effect of individual $i$, $t_{m}$ is the time, and $e_{ijkm}$ is the residual. A separate residual variance was estimated for each month. Other terms are the same as in STM. More details on this model are in [10].

#### Generalized linear mixed model GLMM

To account for the binomial distribution of survival (0/1), we used a generalized linear mixed model with a logit link function. ASReml uses approximate likelihood techniques, of which the limitations are discussed in the Results and discussion section [13]. The model was:

3 $$\begin{aligned} \eta (E(y_{{ikm}} )) & = fixed + \beta _{D} \cdot SNP_{i} \\ & \quad + \;\beta _{I} \cdot \sum\limits_{{j \ne i}}^{{n - 1}} S NP_{{j\left( k \right)}} + a_{i} + cage_{{km}} + PE_{i} , \\ \end{aligned}$$

where $\eta ()$ is the logit link function that links the probability of surviving to the linear predictor, and $E(y_{ikm}$) is the probability of surviving for individual $i$ in cage $k$, with cage mates $j$, at time $m$. The other terms are the same as in STM and RMM.t.

The GLMM includes only a genetic intercept and no regression on time because the non-linear link function takes the change in variance over time into account. Consequently, at the beginning of the recording period, the variance of the survival probabilities can be (near) zero even when $var\left[ {\eta \left( {E\left( {y_{ikm} } \right)} \right)} \right]$ is greater than zero. More details on this model are in [10].

### Model fit

The three models, STM, RMM.t, and GLMM were compared by reviewing -log_10_ *p* values of SNP effects and the size of the inflation factor for *p* values (λ; see below). Pearson correlations between − log_10_ *p* values were calculated to quantify the agreement between the three models.

### Genomic control

A quantile–quantile plot (Q–Q plot) was used to investigate the distribution of the observed *p* values compared to their expected distribution under the null hypothesis that the SNP has no association with the trait. The extent of deviation of the observed distribution from the expected distribution was expressed as λ, where a λ equal to 1 means no deviation [16]. In cases where λ was larger than 1.10, the so-called “genomic control” was applied to avoid spurious associations with the trait by dividing F values by λ before calculating *p* values [16].

Multiple testing was accounted for by controlling the false discovery rate (FDR) using the *q*-value package [17] in R [18]. The FDR is the expected proportion of false-positives among those that were called significant under the distribution of the *p* values. The *q*-value package calculates an FDR based on the distribution of *p* values, which represents the minimum FDR when the SNP effect is called significant, which was set to 0.3. This is a liberal threshold that also reveals suggestive SNP associations with survival time and was chosen because little is known about the background of DGE and IGE for survival time, this study being one of the first large ones.

### Phenotypic and genetic variance explained by SNPs

A backwards elimination method was used to obtain an estimate for phenotypic and genetic variance explained by SNPs. Backwards elimination involved including all direct and indirect SNP effects below the genome-wide FDR threshold (*q* < 0.3) in the model to account for possible linkage disequilibrium (LD) between them, testing their model fit, and dropping the least significant SNP effect. This was repeated until all SNPs reached the FDR threshold. Then, for each remaining SNP, we calculated the genetic variance explained by the SNP following Falconer and Mackay [19] as $V = 2p\left( {1 - p} \right)\alpha^{2}$, with $p$ being the major allele frequency and $\alpha$ is the estimated direct allele substitution effect from the model with all remaining SNPs. LD between fitted SNP effects was not considered in the calculation of $V$.

The proportions of phenotypic variance ($\sigma_{P}^{2}$) and of genetic variance ($\sigma_{A}^{2}$) explained by the direct effects of SNPs were calculated as $\frac{V}{{\sigma_{P}^{2} }}$ and $\frac{V}{{\sigma_{A}^{2} }}$, respectively. The $\sigma_{A}^{2}$ is an estimate of the total genetic variance since group members are related [15].

## Results and discussion

### Model comparison

Genetic parameters were estimated without SNP effects in the model. For all models, variance components were fixed to the estimated values from the corresponding model without SNP effects. The $\sigma_{P}^{2}$ and $\sigma_{A}^{2}$ values are in Table 2. Variance components from RMM.t and GLMM are not presented in Table 2 because they can be translated to the survival time scale, for which estimates are in Table 2 [10]. The total genetic standard deviation ($\sigma_{A} )$ was 24 days for cross W1 * WA, 38 days for cross W1 * WB, and 73 days for cross W1 * WC.

**Table 2:** Estimates of genetic parameters for survival time in three crossbred layer lines using STM

	W1 * WA	W1 * WB	W1 * WC
$\sigma_{A}^{2}$	576 ± 326	1415 ± 583	5310 ± 1386
$\sigma_{c}^{2}$	763 ± 173	1813 ± 287	1832 ± 374
$\sigma_{P}^{2}$	7645 ± 260	10,781 ± 389	15,102 ± 647
$T^{2}$	0.08 ± 0.04	0.13 ± 0.05	0.35 ± 0.08

Estimates of genetic parameters are shown for survival time

$\sigma_{c}^{2} ,\sigma_{A}^{2} ,\sigma_{P}^{2}$, $T^{2}$ are cage variance, genetic variance and phenotypic variance ($\sigma_{P}^{2} = \sigma_{A}^{2} + \sigma_{c}^{2} + \sigma_{e}^{2}$), $T^{2} = \frac{{\sigma_{A}^{2} }}{{\sigma_{P}^{2} }}$ [37], respectively. All variances are in days

There were no evident differences between GWAS results from STM, RMM.t, and GLMM (Tables 3, 4) and [see Additional files 1, 2 and 3]. The same QTL were identified with all three models. Table 3 shows that correlations between − log_10_ *p* values for direct and indirect SNP effects from the three models were higher than 0.9, thus similar SNPs were identified as having weak(-er) or strong(-er) associations in all models. This is in line with findings of Rönnegård et al. [20], who investigated the benefit of using uncensored repeated measures in GWAS in a simulation study with direct effects only. In a design with an equal number of observations per individual, as in our study, they found that the correlation between − log_10_ *p* values between results from a model fitting average phenotypes and a model fitting repeated measures was higher than 0.9 [20]. Rönnegård et al. [20] concluded that a repeated measures model in GWAS was most beneficial when individual phenotypes were very different across time or when the number of observations varied among individuals.

**Table 3:** Pearson correlations between − log_10_ *p* values of the three models for direct and indirect SNP effects for each cross

Effect	Cross	STM-RMM.t	STM-GLMM	RMM.t-GLMM
Direct	W1 * WA	0.98	0.96	0.97
	W1 * WB	0.97	0.93	0.93
	W1 * WC	0.96	0.96	0.93
Indirect	W1 * WA	0.98	0.95	0.96
	W1 * WB	0.98	0.97	0.96
	W1 * WC	0.97	0.97	0.96

All standard errors were less than 0.01

**Table 4:** Inflation factor λ for all crosses and models

Effect	Model	W1 * WA	W1 * WB	W1 * WC
Direct	STM	1.09	1.04	0.91
	RMM.t	1.13	1.18	1.09
	GLMM	1.13	1.02	0.91
Indirect	STM	0.92	1.26	1.10
	RMM.t	0.94	1.43	1.32
	GLMM	0.93	1.22	1.13

All standard errors were less than 0.01

Table 4 shows the inflation factor λ and the corresponding Q–Q plots are in Additional files 1 and 2. There was no clear pattern for λ across models. Moreover, there were no large differences in the number of SNPs with *p* < 0.001 between the three models after genomic control (Note: the aim was to compare models here, not to identify SNPs) [see Additional file 3].

In this study, we used a generalized linear mixed model (GLMM) for GWAS, which was fitted with ASReml [13]. ASReml uses an approximate likelihood technique (the penalized quasi-likelihood), which has not been studied well for hypothesis testing. Gilmour et al. [13] recommend to use GLMM in ASReml with caution. However, when comparing *p* values from the GLMM to those of the other two models, STM and RMM.t, we found that the Q–Q plots from the GLMM behaved well and were similar to those for STM and RMM.t [see Additional files 1 and 2], − log_10_ *p* values from the GLMM were highly correlated with those from STM and RMM.t (Table 3), and Manhattan plots from GLMM showed a similar pattern as those from STM and RMM.t [see Additional files 3 and 4].

Based on these results, we concluded that there was no evidence that RMM.t and GLMM outperformed STM. Thus, in the remainder of this paper, we will only show results from STM.

### GWAS results (STM)

**Fig. 1:**
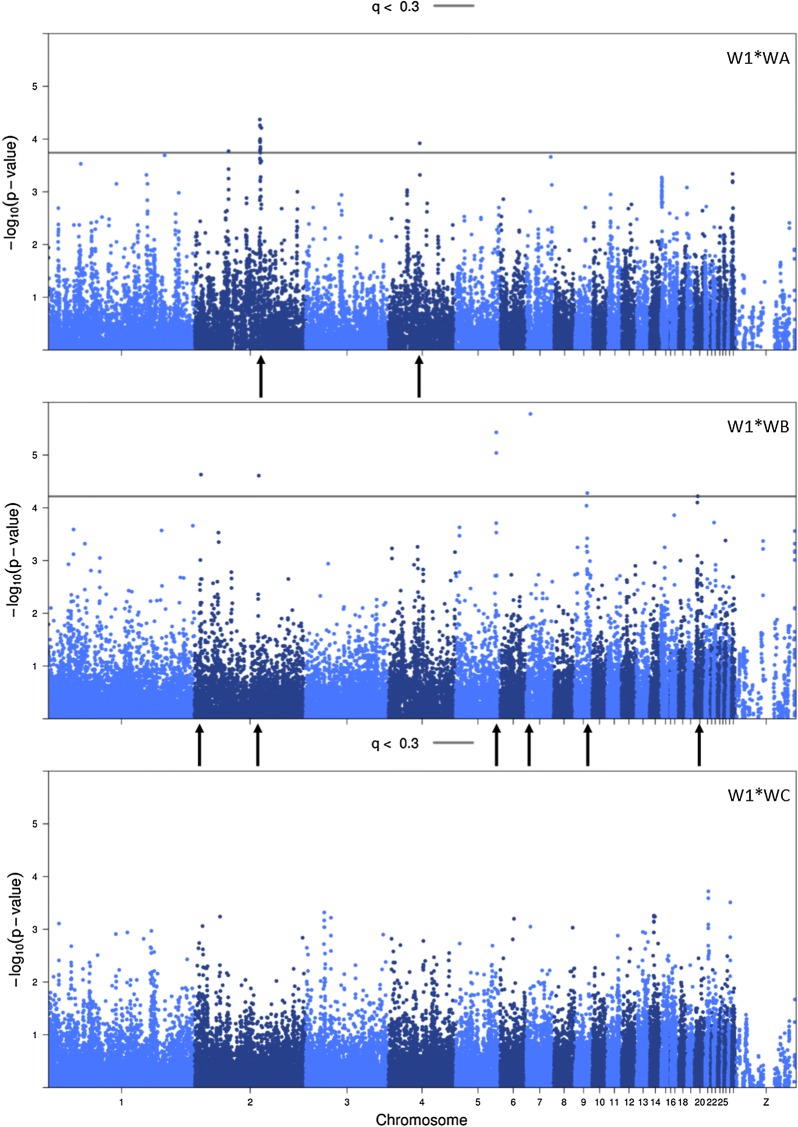
Manhattan plots of direct SNP effects for crosses W1 * WA, W1 * WB, and W1 * WC. FDR threshold was 0.30 (solid line). If no SNP reached the FDR-threshold, the threshold could not be estimated (Panel 3). Locations of SNPs with *q* < 0.3 are indicated with an arrow

**Fig. 2:**
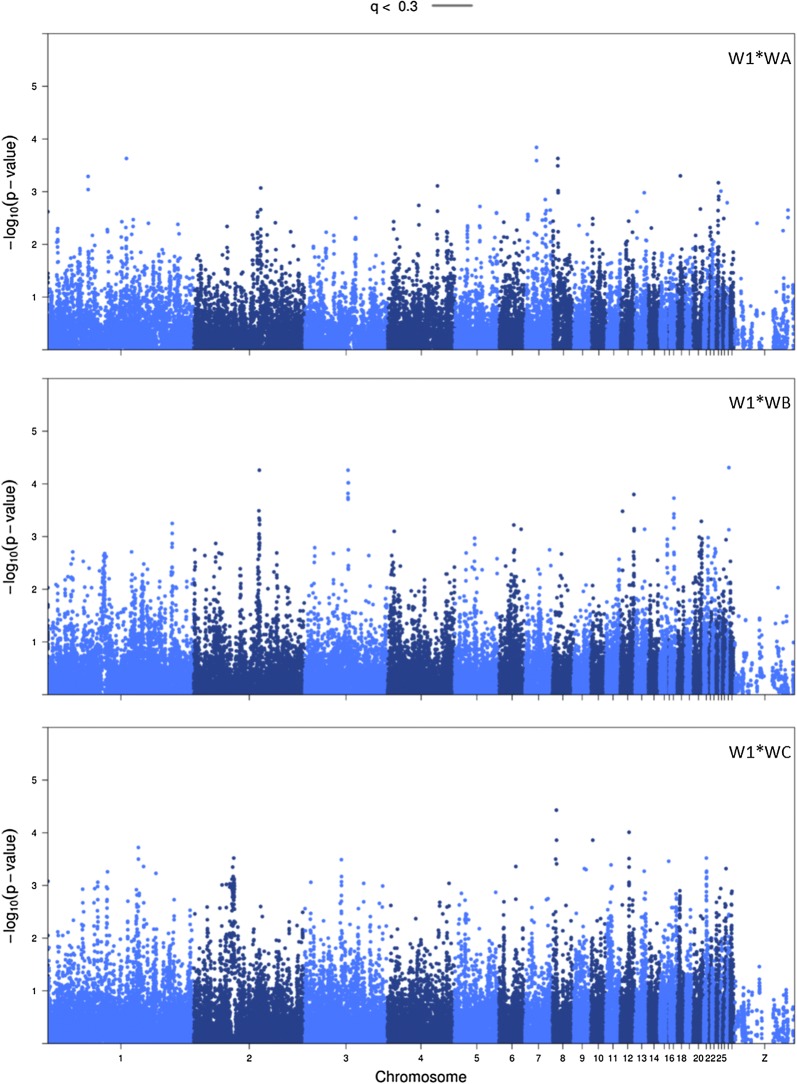
Manhattan plots of indirect SNP effects for crosses W1 * WA, W1 * WB, and W1 * WC. FDR threshold was 0.30 (solid line). If no SNP reached the FDR-threshold, the threshold could not be estimated (Panels 1, 2 and 3)

Figures 1 and 2 show the Manhattan plots for the direct and indirect SNP effects for the three crosses, respectively. Several SNPs were associated with direct effects for survival time at *q* < 0.3 in cross W1 * WA and W1 * WB, but none in cross W1 * WC. In none of the crosses, SNPs were associated with indirect effects for survival time at *q* < 0.3.

In cross W1 * WA, 17 SNPs were associated with direct effects for survival time at *q* < 0.3. Of these, one SNP was on chromosome 4 at 42 Mb. The remaining 16 SNPs were on chromosome 2, with one SNP at 46 Mb, and 15 SNPs in the region between 87 and 89 Mb. The latter 15 SNPs were in high LD with most pairwise *r*^*2*^ higher than 0.9. After backwards elimination, one SNP (rs317294317) remained in the model (*q* < 0.3, corresponding to *p* < 2.05E−5, Table 5). This SNP is an intron variant at 88 Mb on chromosome 2, had an estimated effect of 22 days, and explained 3% of the phenotypic variance ($\sigma_{p}^{2}$), and 36% of the total genetic variance ($\sigma_{A}^{2}$).

**Table 5:** Significant (*q* < 0.30) direct and indirect SNP effects, their location, minor allele frequency (MAF), and estimated effect size α, based on model STM

Effect	Cross	SNP	Chr	Position (kbp)	MAF	α (days)	*q*-value	V (days^2^)	% of $\sigma_{P}^{2}$ explained	% of $\sigma_{{A_{T} }}^{2}$ explained
Direct	W1 * WA	rs317294317	2	88,120	0.34	22 ± 5	0.29	209	2.7	36.3
Direct	W1 * WB	rs313098101	2	8799	0.32	20 ± 5	0.16	167	1.5	11.8
		rs31610924	5	54,321	0.35	16 ± 4	0.06	122	1.1	8.6
		rs312488612	7	5646	0.47	35 ± 8	0.05	627	5.8	44.3
		rs14677635	9	17,018	0.22	26 ± 6	0.28	229	2.1	16.2

In cross W1 * WB, seven SNPs were associated with direct effects for survival time at *q* < 0.3. These were located on chromosome 2 (at 9 and 86 Mb), chromosome 5 (two SNPs at 54 Mb), chromosome 7 (at 6 Mb), chromosome 9 (at 17 Mb), and chromosome 20 (at 20 Mb). After backwards elimination, four SNPs remained in the model (*q* < 0.3, corresponding to *p* < 2.84E−05, Table 5). Estimated effect sizes ranged from 16 to 35 days, with rs31610924 having the smallest effect size and rs312488612 having the biggest effect size. The SNPs explained 1–6% of $\sigma_{p}^{2}$ and 9–44% of $\sigma_{A}^{2}$.

Biscarini et al. [6] was the first to investigate the genetic architecture of both direct and indirect genetic effects of plumage condition in laying hens. They found 11 direct associations and 81 indirect associations between SNPs and plumage condition. Our study did not confirm the large number of indirect SNP associations, although it is one of the first large GWAS that includes both DGE and IGE. We analyzed survival time, which reflects the final stage of cannibalism, while plumage condition is recorded before the end stage. This could partly explain the difference in results with Biscarini et al. [6].

#### Candidate genes

*Chromosome 2* In cross W1 * WA, SNP rs317294317 remained in the model after backwards elimination. This SNP is an intron variant on chromosome 2. The associated gene is *GABBR2* (88.0–88.4 Mb; Fig. 1). A clear peak is visible for direct SNP effects in this region for cross W1 * WA. Moreover, we observed a clear peak in the same region for indirect SNP effects in cross W1 * WB—although not significant at *q* < 0.3 after genomic control (Fig. 2). The favorable allele was the same for the direct SNP effect in cross W1 * WA and the indirect SNP effect in cross W1 * WB. This allele has a positive effect both on the survival of the individual itself and on the survival time of its group mates. The *GABBR2* gene was found to be associated with both direct phenotypes and behavioral phenotypes [21–25].

The *GABBR2* gene encodes a receptor for gamma-aminobutyric acid (GABA), which plays an important role in the regulation of neurotransmitters in the brain. GABA is an inhibitor of neuronal activity and plays an important role in physiological and behavioral stress response in many species [21–25]. Zhang et al. [25] for example, found that the level of GABA affects the performance and physical condition in Roman laying hens under heat stress. Furthermore, Poshivalov [22] found that the level of GABA was associated with a change in state of aggressiveness and sociability towards conspecifics in *Mus musculus*.

In addition to GABA, serotonin and dopamine are also important neurotransmitters and are known to be associated with several behavioral disorders in a variety of species. Moreover, several studies have reported a link between the serotonergic, dopaminergic, and GABAergic pathways [26–28]. Biscarini et al. [6] investigated the genetic architecture of direct and indirect genetic effects of plumage condition in laying hens and found a SNP in the *HTR2C* gene, which is involved in the serotonergic system, that was associated with indirect genetic effects. Another study on aggression in chickens revealed a role of the dopaminergic system [29]. Moreover, Bolhuis et al. [30] investigated the effects of group selection on survival on serotonin levels and suggested that the level of serotonin may be linked to the development of cannibalism. Indeed, the study of Flisikowski et al. [31] concluded that genomic regions related to the dopaminergic and serotonergic systems were associated with feather pecking behavior in laying hens. Thus, our results support those from two other GWAS that focused on a feather pecking related trait in chickens.

*PTPRN2* In cross W1 * WB, SNP rs313098101 remained in the model after backwards selection. This SNP is an intron variant on chromosome 2. The associated gene is *PTPRN2* (9 Mb; Fig. 1), which encodes a receptor for protein tyrosine phosphatase and is associated with several disease phenotypes [32]. However, it is likely that the association of SNP rs313098101 with direct effects for survival time is a false positive association, given that no clear peak is visible in this region.

#### Percentage of genetic variance explained

The contribution of DGE associated SNPs with *q* < 0.3 to the total genetic variance after backwards elimination was large and summed up to 36% for cross W1 * WA and to 81% for cross W1 * WB (Table 5). These genetic variances explained by the SNPs are probably overestimated because of the Beavis effect, i.e., when many effects are tested for significance and only those below the defined significance threshold are considered, SNP estimates tend to be overestimated [33]. This especially occurs when the power of the study is low.

Moreover, the proportion of genetic variance explained by SNPs associated with direct effects was calculated as $\frac{V}{{\sigma_{A}^{2} }}$, where $V$ is the variance explained by the SNPs, and $\sigma_{A}^{2}$ is an estimate of the total genetic variance. The latter is the sum of variances due to direct and indirect genetic effects, along with a component due to their covariance [15]. Previous research reported negative genetic correlations between direct and indirect effects for survival time in crossbred layers [2]. If the correlation between direct and indirect genetic effects is strongly negative, the total $\sigma_{A}^{2}$ may be smaller than the variance due to the direct effects, which could partly be the reason for the possible over-estimation of the proportion of $\sigma_{A}^{2}$ explained by direct SNP effects in this study. We are interested in the proportion of direct genetic variance that is explained by DGE associated SNPs. However, with cages composed of families, an animal model with direct genetic effects only will also pick up many indirect genetic effects [14]. The variance due to direct genetic effects alone was, thus, unknown.

### Power

Statistical power of a GWAS depends on several factors such as the number of observations, relatedness among individuals, allele frequency, level of linkage disequilibrium, and the statistical model [34]. To get an estimation of the power of our data for GWAS, we calculated power by assuming a number of true direct SNP effects from 1 to 18 days at allele frequencies ranging from 0 to 1, given the population specific parameters of the crosses (*N* and $\sigma_{P}^{2}$; Table 2) and significance threshold *q* < 0.3 (~ 4 standard deviations from the mean; [see Additional file 4]). We assumed a normal distribution. Theoretical findings as presented in Additional file 4 were supported by the empirical evidence obtained from this study. The results suggest that the statistical power of the GWAS was highest for cross W1 * WA and lowest for cross W1 * WC. True direct effects, at an allele frequency of 0.5, had to be at least 15 days for W1 * WA, 16 days for W1 * WB, and 21 days for W1*WC in order to be detected with reasonable probability (power ~ 0.8). True indirect effects, at an allele frequency of 0.5, had to be at least 3.5 days for W1 * WA, 4 days for W1 * WB, and 5 days for W1 * WC in order to be detected with reasonable probability (power ~ 0.8). The number of observations in cross W1 * WC was much smaller compared to the other two crosses, which may explain why the power of this cross was lowest.

Theoretical power to detect indirect SNP effects was higher than power to detect direct SNP effects. This is due to the multiplication by the number of group mates for indirect SNP effects. It is, therefore, possible that SNP with a lower MAF can be detected at *q *< 0.3 as associated with IGE rather than DGE. In addition, the contribution of IGE to the total heritable variance of survival time in laying hens is often larger than the contribution of DGE [2, 3, 35]. Moreover, in the absence of kin selection, IGE are less exposed to natural selection than DGE [5, 36], and some loci may therefore have large effects. Nevertheless, no IGE associated SNPs were found at *q* < 0.3. Perhaps the level of mortality in crosses W1 * WA and W1 * WB (22 and 25%) was insufficient for detection of IGE associated SNPs, i.e., with lower mortality, fewer individuals will die due to cannibalism and less indirect genetic variance will be available for SNP detection. For cross W1 * WC, theoretical power for GWAS was lower than for W1 * WA and W1 * WB and more individuals died before blood sampling. These hens were given an average genotype while having an extreme phenotype, which may explain why no direct and indirect SNP associations were found in cross W1 * WC.

## Conclusions

This is one of the first large studies that investigates the genetic architecture of a trait by considering both direct and indirect genetic effects. Our results indicate that the same QTL were identified using either a linear mixed model of survival time or models of repeated binomial survival (0/1). Although the power was relatively low, and many SNP associations may have not been detected, our results revealed a link of the GABAergic system with direct and indirect genetic effects for survival time in crossbred layers. The associated gene was *GABBR2*. This supports existing evidence of the involvement of GABA in the development of abnormal behaviors.

## Additional files

**Additional file 1.** QQ-plots of direct SNP effects for STM, RMM.t, and GLMM.

**Additional file 2.** QQ-plots of indirect SNP effects for STM, RMM.t, and GLMM.

**Additional file 3.** Number of direct and indirect SNP effects with p < 0.001 for all crosses and models after genomic control.

**Additional file 4.** Power of direct and indirect SNP effects.
