# Supplementary figures and images for: The genetic architecture of socially-affected traits: a GWAS for direct and indirect genetic effects on survival time in laying hens showing cannibalism
Source: Genet Sel Evol. 2018 Jul 23;50:38. doi: 10.1186/s12711-018-0409-7 (PMC6057005; doi:10.1186/s12711-018-0409-7)

**Appendix I – QQ-plots of direct SNPs for STM, RMM.t, and GLMM.**

| *Cross* | *STM* | *RMM.t* | *GLMM* |
| --- | --- | --- | --- |
| *W1*WA* | 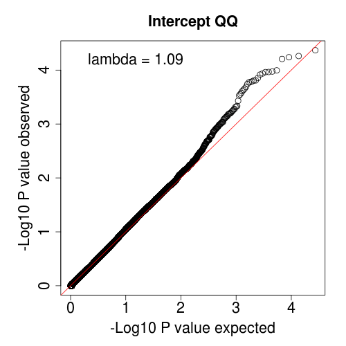 | 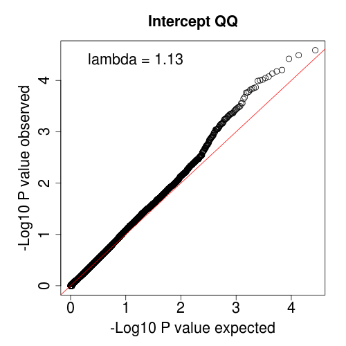 | 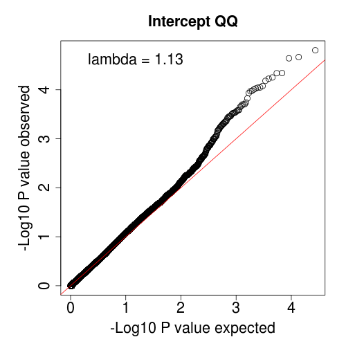 |
| *W1*WB* | 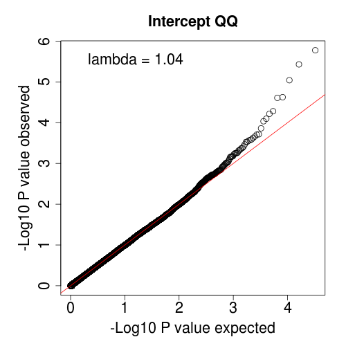 | 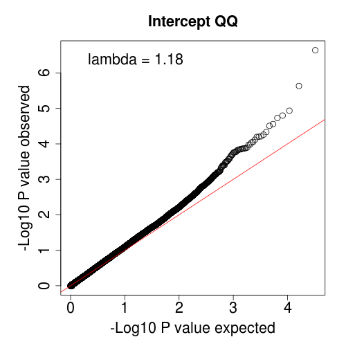 | 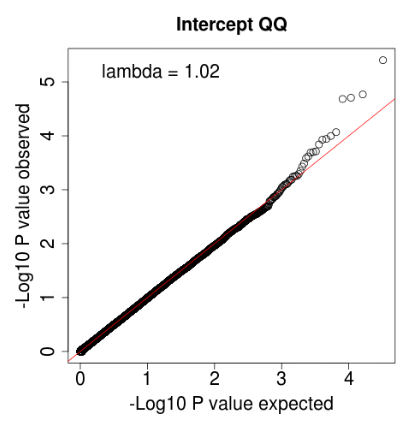 |
| *W1*WC* | 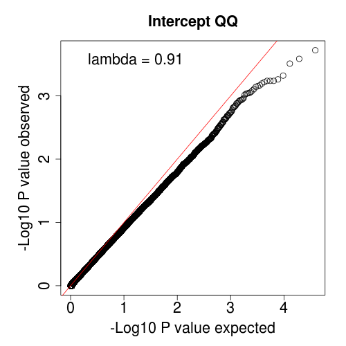 | 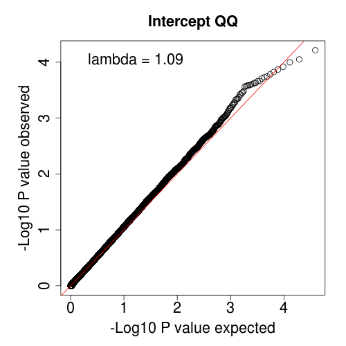 | 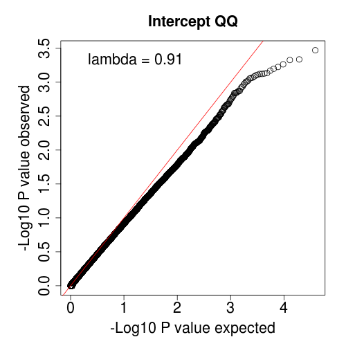 |

Supplement: Supplementary file 1 — Additional file 1. QQ-plots of direct SNP effects for STM, RMM.t, and GLMM. [file 12711_2018_409_MOESM1_ESM.docx]

**Appendix II – QQ-plots of indirect SNPs for STM, RMM.t, and GLMM.**

| *Cross* | *STM* | *RMM.t* | *GLMM* |
| --- | --- | --- | --- |
| *W1*WA* | 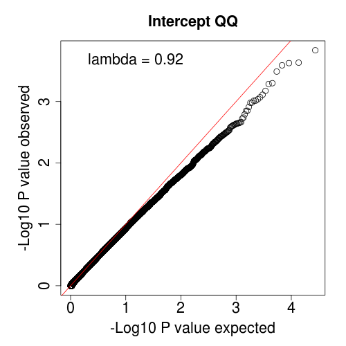 | 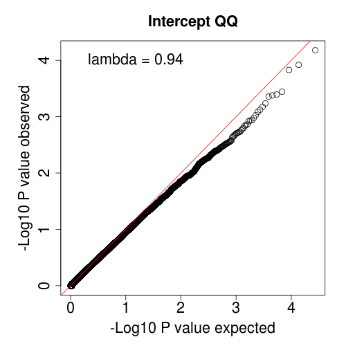 | 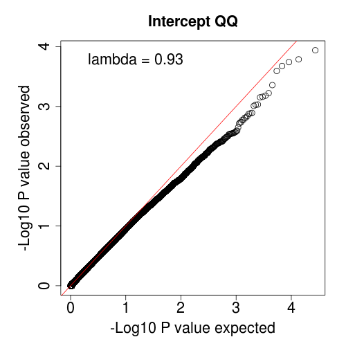 |
| *W1*WB* | 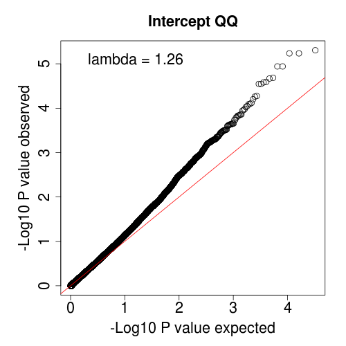 | 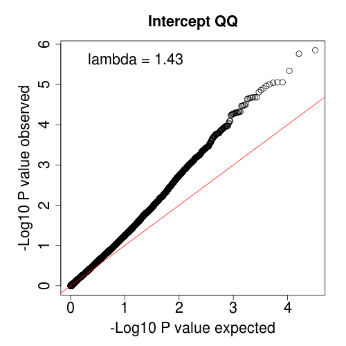 | 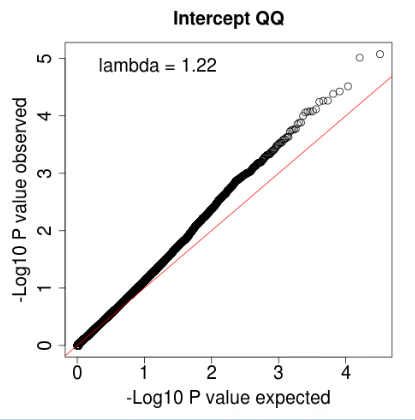 |
| *W1*WC* | 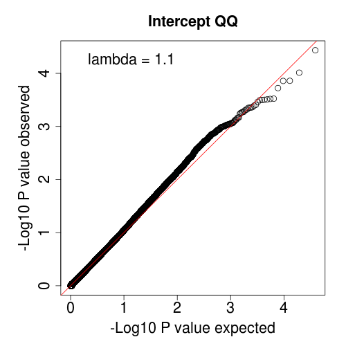 | 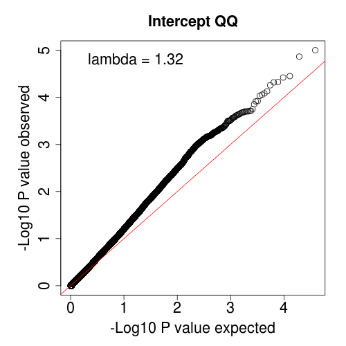 | 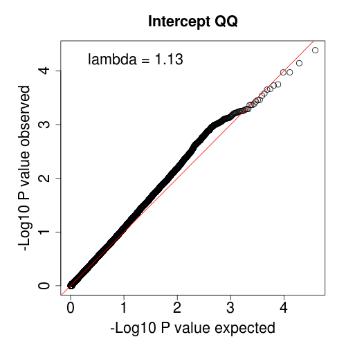 |

Supplement: Supplementary file 2 — Additional file 2. QQ-plots of indirect SNP effects for STM, RMM.t, and GLMM. [file 12711_2018_409_MOESM2_ESM.docx]
