# Supplementary material for: The genetic architecture of socially-affected traits: a GWAS for direct and indirect genetic effects on survival time in laying hens showing cannibalism
Source: Genet Sel Evol. 2018 Jul 23;50:38. doi: 10.1186/s12711-018-0409-7 (PMC6057005; doi:10.1186/s12711-018-0409-7)
Supplement: Supplementary file 3 — Additional file 3. Number of direct and indirect SNP effects with p < 0.001 for all crosses and models after genomic control. [file 12711_2018_409_MOESM3_ESM.docx]

**Appendix III - Number of direct and indirect SNPs with *p*<0.001 for all crosses and models after genomic control**

| Effect | Model | W1*WA | W1*WB | W1*WC |
| --- | --- | --- | --- | --- |
| Direct | STM | 236 | 278 | 345 |
|  | RMM.t | 226 | 283 | 332 |
|  | GLMM | 237 | 245 | 330 |
| Indirect | STM | 226 | 356 | 374 |
|  | RMM.t | 236 | 355 | 376 |
|  | GLMM | 213 | 413 | 361 |
