# Supplementary material for: The genetic architecture of socially-affected traits: a GWAS for direct and indirect genetic effects on survival time in laying hens showing cannibalism
Source: Genet Sel Evol. 2018 Jul 23;50:38. doi: 10.1186/s12711-018-0409-7 (PMC6057005; doi:10.1186/s12711-018-0409-7)
Supplement: Supplementary file 4 — Additional file 4. Power of direct and indirect SNP effects. [file 12711_2018_409_MOESM4_ESM.docx]

**Appendix IV – Power of direct and indirect SNP effects**

To get an impression of the power in our data we calculated the power assuming a true SNP effect in days at allele frequencies ranging from 0-1, given the population specific parameters of the crosses (**Table 1**), and with an acceptance threshold of ~4 standard deviations from the mean.

Assuming a simple model for direct SNP effects: $y_{i}=\mu+b_{D}\cdot{SNP}_{i}+e_{i}$,

The SE of direct SNP effect $b_{D}$ is given by:

$$SE\left( b_{D} \right)=\frac{\sigma_{e}}{\sqrt{2Np(1-p)}},$$

With $SE\left( b_{D} \right)$ the standard error of direct SNP effect $b_{D}$, $\sigma_{e}$ being the residual standard deviation, *N* the sample size, and *p* the allele frequency.

The power was obtained from:

$$PWR\left( b_{D} \right)=1-pnorm(4-\frac{\left| b_{D} \right|}{SE\left( b_{D} \right)})$$

Assuming a simple model for indirect SNP effects: $y_{i}=\mu+b_{I}\cdot\sum_{i\neq j}^{n-1} {SNP}_{j}+e_{i}$,

The SE of indirect SNP effect $b_{I}$ is given by:

$$SE\left( b_{I} \right)=\frac{\sigma_{e}}{\sqrt{{(n-1)}^{2}2Np(1-p)}},$$

With $SE\left( b_{I} \right)$ the standard error of indirect SNP effect $b_{I}$, and *n* the number of group members.

The power was obtained from:

$$PWR\left( b_{I} \right)=1-pnorm(4-\frac{\left| b_{I} \right|}{SE\left( b_{I} \right)})$$

1. **Graphs of power at allele frequencies 0-1.00 for direct SNP effects b**

*
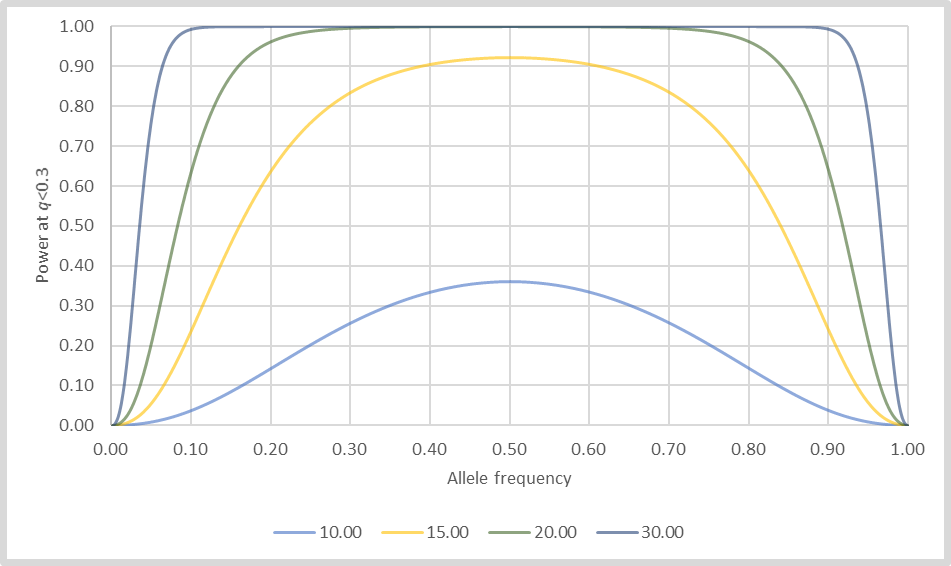
*

**Power of detecting direct SNP effect b (ranging from 0-30 days) for a range of allele frequencies (0-1.00) for cross W1*WA.**

*
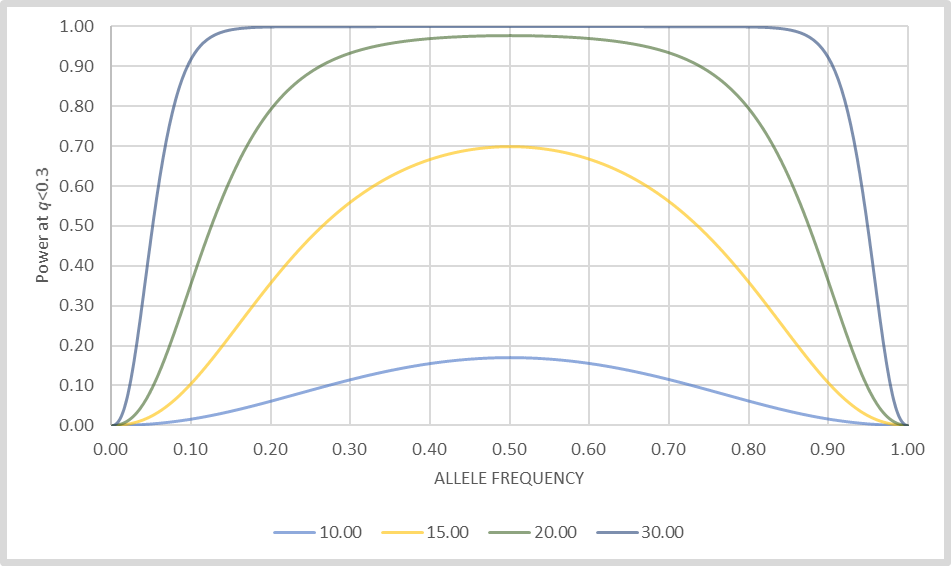
*

**Power of detecting direct SNP effect b (ranging from 0-30 days) for a range of allele frequencies (0-1.00) for cross W1*WB.**


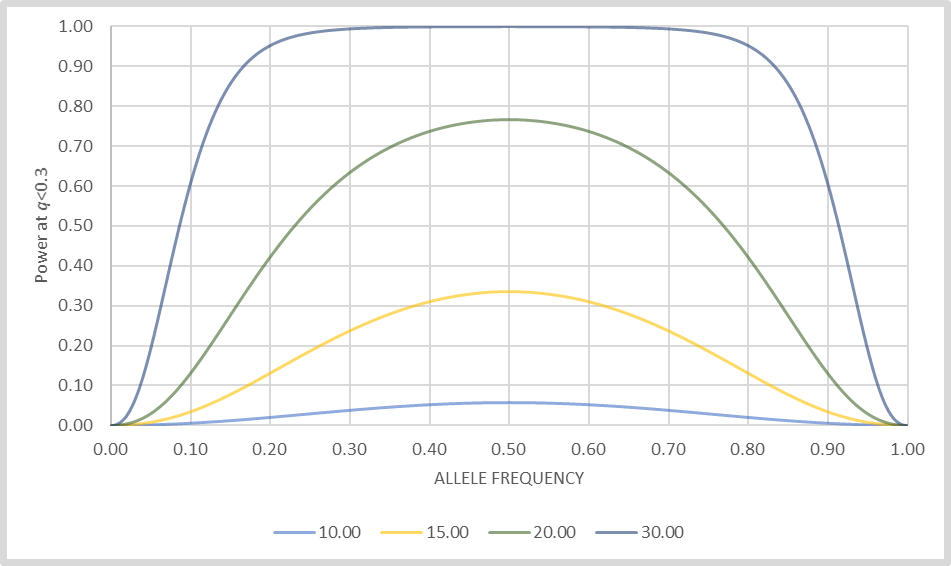


**Power of detecting direct SNP effect b (ranging from 0-20 days) for a range of allele frequencies (0-1.00) for cross W1*WC.**

1. **Graphs of power at allele frequencies 0-1.00 for indirect SNP effects b**

**
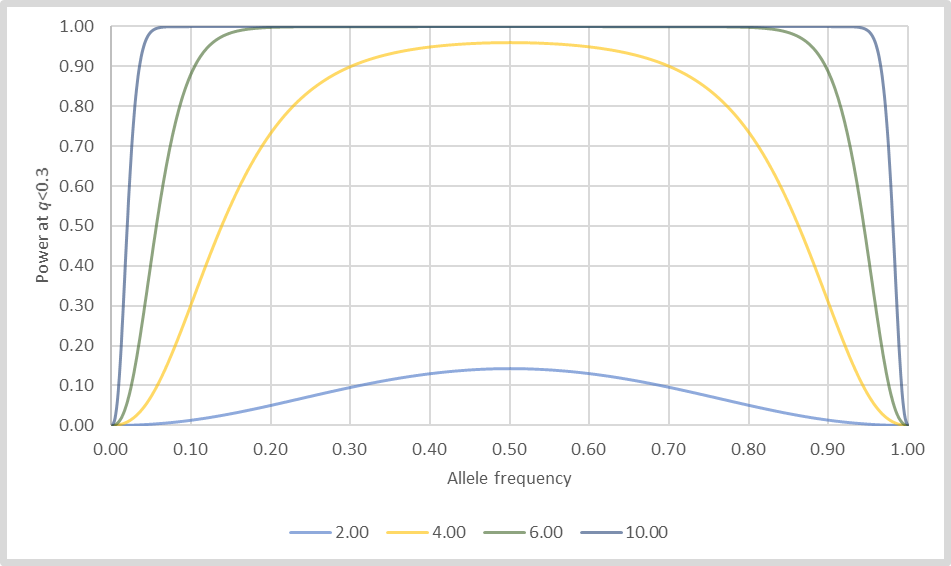
**

**Power of detecting indirect SNP effect b (ranging from 0-10 days) for a range of allele frequencies (0-1.00) for cross W1*WA.**

**
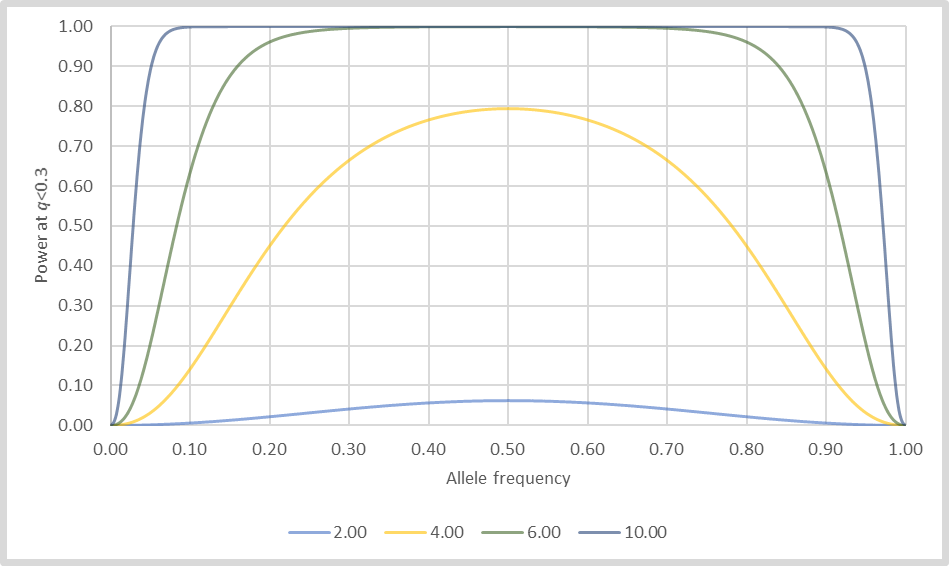
**

**Power of detecting indirect SNP effect b (ranging from 0-10 days) for a range of allele frequencies (0-1.00) for cross W1*WB.**

**
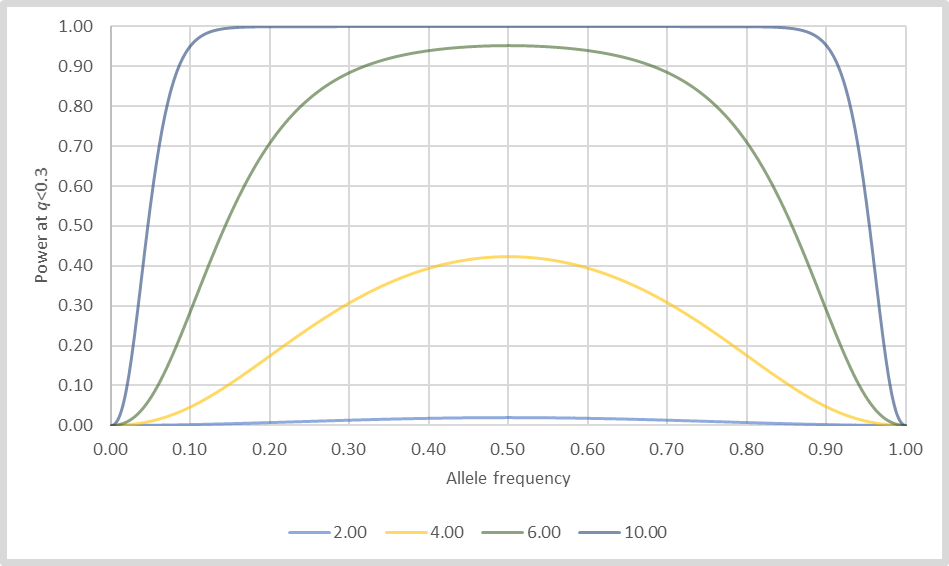
**

**Power of detecting indirect SNP effect b (ranging from 0-30 days) for a range of allele frequencies (0-1.00) for cross W1*WC.**
